# Inhibition of Bruton's tyrosine kinase restricts neuroinflammation following intracerebral hemorrhage

**DOI:** 10.7150/thno.101024

**Published:** 2025-01-01

**Authors:** Hongying Hao, Tingyu Yin, Tuo Li, Xu Zhou, Honglei Ren, Mingming Liu, Huachen Huang, Caiyun Qi, Yuwen Xiu, Wenjin Qiu, Danni Wang, Mengxuan Shi, Xiaoying Wang, Aaron S. Dumont, Qiang Liu

**Affiliations:** 1 Department of Neurology, Tianjin Neurological Institute, Tianjin Institute of Immunology, State Key Laboratory of Experimental Hematology, International Joint Laboratory of Ocular Diseases, Ministry of Education, Haihe Laboratory of Cell Ecosystem, Laboratory of Post-Neuroinjury Neurorepair and Regeneration in Central Nervous System Tianjin & Ministry of Education, Tianjin Medical University General Hospital, Tianjin 300052, China.; 2 Department of Neurosurgery, Tianjin Huanhu Hospital, Tianjin 300350, China.; 3 Tulane Center for Clinical Neurosciences, Department of Neurosurgery and Neurology, Tulane University School of Medicine, New Orleans, LA 70112, USA.; 4 Department of Neurosurgery, The Affiliated Hospital of Guizhou Medical University, Guiyang 550001, Guizhou Province, China.

**Keywords:** Intracerebral hemorrhage, Neuroinflammation, Microglia, Bruton's tyrosine kinase, Ibrutinib.

## Abstract

**Background:** Intracerebral hemorrhage (ICH) is a devastating form of stroke with a lack of effective treatments. Following disease onset, ICH activates microglia and recruits peripheral leukocytes into the perihematomal region to amplify neural injury. Bruton's tyrosine kinase (BTK) controls the proliferation and survival of various myeloid cells and lymphocytes. However, the role of BTK in neuroinflammation and ICH injury remains poorly understood.

**Methods:** Peripheral blood samples were collected from ICH patients and healthy controls to measure BTK expression profile in immune cell subsets. C57BL/6 mice were used to measure BTK expression and the activity of immune cell subsets following ICH induction. Neurological tests, brain water content, Evans blue leakage, MRI were used to assess the therapeutic effects of ibrutinib on ICH injury. Flow cytometry was used to investigate immune cell infiltration and microglial activity. Microglia were depleted using a CSF1R inhibitor PLX5622. Gr-1^+^ myeloid cells and B cells were depleted using monoclonal antibodies. Microglia-like BV2 cells were cultured to test the effects of BTK inhibition on these cells.

**Results:** In humans and mice, we found that BTK was remarkably upregulated in myeloid cells after ICH. Inhibition of BTK using ibrutinib led to reduced neurological deficits, perihematomal edema, brain water content and blood-brain barrier disruption. BTK inhibition suppressed the inflammatory activity of microglia and brain infiltration of leukocytes. In contrast, BTK inhibition did not alter the counts of peripheral immune cells other than B cells. Further, the depletion of microglia or Gr-1^+^ myeloid cells ablated the protective effects of BTK inhibition against ICH injury. Notably, the depletion of B cells did not alter the protective effects of BTK inhibition against ICH injury. This suggests that the benefit of BTK inhibition in ICH mainly involves its impact on microglia and Gr-1^+^ myeloid cells.

**Conclusion:** Our findings demonstrate that BTK inhibition attenuates neuroinflammation and ICH injury, which warrants further investigation as a potential therapy for ICH.

## Introduction

Intracerebral hemorrhage (ICH) is a devastating stroke subtype with high mortality and disability 1. In patients with critical life-threatening conditions, hematoma clearance by surgical evacuation or clot aspiration reduces mortality 2 and improves neurological outcome in a number of clinical studies and meta-analyses 3. The effectiveness of pharmacological approaches such as hyperosmolar therapy and iron chelation remains uncertain and awaits further investigation in ICH patients. As such, ICH remains the least treatable stroke subtype, highlighting an urgent yet unmet need for effective management of ICH.

ICH activates the immune system and elicits neuroinflammation that exacerbates perihematomal edema (PHE) formation, mass effect and cell death 4-6. Microglia are among the first responders to ICH and engage in intimate crosstalk with other brain cells and infiltrating leukocytes to amplify ICH injury 7. Among the major brain-infiltrating leukocyte subsets, lymphocytes and myeloid cells such as monocytes and neutrophils are found in the cerebrospinal fluid and perihematomal brain tissue obtained from ICH patients and contribute to PHE expansion 8, 9. These findings suggest that targeting microglia and brain-infiltrating leukocytes to counteract detrimental neuroinflammation may improve the prognosis of ICH.

As a non-receptor-bound intracellular signaling protein, activation of Bruton's tyrosine kinase (BTK) is vital for the proliferation and survival of B lymphocytes 10-13 and myeloid cells, including monocytes, neutrophils, and macrophages 14-16 as well as microglia that reside in the central nervous system (CNS) 17, 18. The distribution and function of BTK suggest its involvement in the initiation and evolution of tissue inflammation 19, 20. Accumulating evidence suggests that BTK inhibition can suppress neutrophil activation 21, orchestrate myeloid cell recruitment and modulate inflammatory responses 22-24. However, the precise role of BTK in neuroinflammation and the impact of BTK inhibition on ICH injury remain poorly understood.

In this study, we examined the impact of BTK inhibition on neuroinflammation and ICH injury using an irreversible and selective small-molecule inhibitor of BTK, ibrutinib 25, 26. In two mouse models of ICH, we found that BTK inhibition suppressed the inflammatory activity of microglia and brain infiltration of leukocytes, leading to reduced neuroinflammation and ICH injury. The benefits of BTK inhibition in ICH involved its impact on microglia and peripheral Gr-1^+^ myeloid cells, i.e. neutrophils and monocytes.

## Methods

### Animals

All animal experiments were conducted in accordance with the National Institutes of Health Guide for the Care and Use of Laboratory Animals in China and approved by the Committee on the Ethics of Animal Experiments of Tianjin Neurological Institute, Tianjin Medical University General Hospital, Tianjin, China. The research adheres to the requirements of the ARRIVE (Animal Research: Reporting *in vivo* Experiments) statement. Male C57BL/6 mice that were eight weeks old (weighing 22-24 g) were bought from the Vital River Corporation (Beijing, China). Mice were randomly assigned to each experimental group. Mice were housed in the vivarium facilities under a standardized light-dark cycle in a pathogen-free environment with unlimited access to food and water.

### Human peripheral blood samples

Patient studies were carried out in conformity with the Helsinki Declaration. The Ethics Committees of Tianjin Medical University General Hospital have given their approval for the enrollment of human subjects. All individuals provided their informed consent at the time of the enrollment. Peripheral blood samples for flow cytometry were collected from 10 male patients with ICH and 10 male healthy controls. Regarding the recruited subjects' ages, there was no statistical difference (ICH vs. Control: 56.5 ± 5.4 vs. 55.3 ± 4.6 years, P = 0.605). Inclusion standards for healthy controls: 1) The age of the subjects ranged from 45 to 65 years old; 2) Basic laboratory tests were normal and generally healthy overall. Inclusion criteria for patients with ICH: 1) The individuals were 45 to 65 years old, and 2) no immune-modulating medication was administered to them. Patients with acute myocardial infarction, heart failure, liver conditions, tumors, peripheral autoimmune diseases, hematological conditions, any hemodynamic instability at the time of consent, any infection before the onset of the disease, concurrent use of antineoplastic or immune-modulating therapies, and others were excluded.

### Experimental design and drug administration

To determine whether BTK expression was increased following ICH, ICH was induced by injection of bacterial collagenase. An identical volume of sterile saline injection was given to sham mice. On the third day following ICH, cells were collected for flow cytometry. Likewise, peripheral blood samples from patients were taken for flow cytometry examination on the third day following ICH. Healthy controls' peripheral blood samples were taken simultaneously for flow cytometric analysis.

To determine the effects of BTK inhibitor ibrutinib (PCI-32765, HY-10997, MedChemExpress, Monmouth Junction, NJ, USA) in mice after ICH. ICH was induced by injection of bacterial collagenase or autologous blood. Mice received daily intraperitoneal (i.p.) injections of ibrutinib (10 mg/kg) or an equal volume of vehicle (10% DMSO, 40% PEG300, 5% Tween-80, 45% saline) for 3 consecutive days starting 12 hours after ICH induction. The therapeutic impact of ibrutinib was assessed using neurological tests, brain water content measurements, Evans blue leakage, hematoma volume measurements, and edema volume measurements.

To deplete microglia, PLX5622 (Selleckchem, Houston, TX) was formulated in AIN-76A standard chow at 1.2 g PLX5622 per kilogram of diet to study the potential mechanism of ibrutinib in neuroprotection against ICH. Before ICH induction, six-week-old mice were fed either control chow or chow with PLX5622 for a period of 14 consecutive days 27. Neurological tests were conducted on days 1 and 3 following ICH. At day 3 following ICH, samples were taken for flow cytometry and the content of brain water. To deplete Gr-1^+^ myeloid cells *in vivo*, 250 μg of anti-mouse Ly6G/Ly6C (Gr-1) monoclonal antibody (Clone ID: RB6-8C5; BioLegend, San Diego, CA) were injected intraperitoneally (i.p.) into each animal one day before and one day after ICH surgery. Neurological tests were conducted on days 1 and 3 following ICH. At day 3 following ICH, samples were taken for flow cytometry and the amount of brain water. Anti-mouse CD20 monoclonal antibody (clone ID: SA271G2; BioLegend, San Diego, CA) was administered intravenously (i.v.) at 10 mg/kg 3 days before ICH induction to deplete B cells. Neurological tests were conducted on days 1 and 3 following ICH. At day 3 following ICH, samples were then collected for flow cytometry and brain water content analysis.

### ICH induction

ICH was induced in mice by injecting bacterial collagenase or autologous blood, as previously described^28^. Briefly, mice were anesthetized by 3% isoflurane inhalation and maintained by 1% isoflurane during surgery. These mice were subsequently fixed on a stereotactic frame. Following a midline scalp incision, the right basal ganglia (0.5 mm anterior and 2.3 mm lateral of the bregma, and 3.5 mm depth below the surface of the skull) was infused with 0.0375 U bacterial collagenase (type IV-S; Sigma-Aldrich, St. Louis, MO) in 0.5 μl saline at a rate of 0.5 μl/min. The autologous blood injection model used a two-step injection method. First, 5 ul autologous blood was infused (3 mm depth below the surface of the skull), then 25 ul autologous blood was infused (3.5 mm depth below the surface of the skull), and the injection was completed. An identical volume of saline was injected into sham mice. Super Glue (Loctite, Westlake, OH) was used to close the burr hole. Throughout the experimental and early recovery periods, rectal temperature was measured and kept at 37.0 ± 0.5℃. In this study, 574 mice in total were used, and 456 of these mice were subjected to ICH surgery. A total of 30 mice died following ICH surgery. The overall mortality rate was ~6.6% (30/456).

### Neurological assessment

At least two investigators who were blinded to the experimental groups assessed the neurological function following ICH. The modified Neurologic Severity Score (mNSS), corner turn test, and rotarod test were performed 28-31. The scale for the mNSS is 0 to 18, with scores ranging from 13 to 18 denoting severe injury, 7 to 12 denoting moderate injury, and 1 to 6 denoting mild injury. During the corner turn test, mice were allowed to enter a 30° corner formed by two mirrors and were required to turn to the left or right in order to exit the corner. A score was calculated as follows: the number of right turns/10 trials×100%. Mice were used in the rotarod test after being trained for 3 days prior to ICH induction, and then were positioned in the center position on the accelerating rotating rod (acceleration from 5 rpm to 40 rpm in 90 seconds, then 40 rpm for up to 5 min). Rotarod test results ranged from 0 to 300 sec. Blinded investigators recorded the fall latency of each mouse. The average incubation period of three consecutive tests in a day represents the motor performance of mice. The test was performed on day 1 and day 3 after ICH.

### Brain water content measurement

We evaluated brain water content as previously reported 28. In brief, mice were euthanized at day 3 following ICH. The wet weights of the contralateral and ipsilateral hemispheres, as well as the cerebellum (an internal control), were individually weighed on an analytical balance, dried for 24 h at 100 °C, and then measured again to get dry weights. Mouse brains were dissected without perfusion. The percentage of brain water was calculated as (wet weight minus dry weight)/wet weight×100%.

### Assessment of BBB permeability

As previously described 28, 32, Evans blue dye (Sigma Aldrich, St. Louis, MO, USA) extravasation assays were performed at day 3 following ICH to assess BBB permeability. Mice received 100 µL of 2% Evans blue through the tail vein. Two hours after injection, the contralateral and ipsilateral hemispheres were obtained and weighed separately after euthanasia and PBS perfusion, then homogenized in 1 mL formamide (Aladdin, Shanghai, China) per 0.1 g brain tissue before incubation at 60 ◦C overnight. The brain homogenates were centrifuged at 16,000 rpm for 30 min. The Evans blue in the supernatant was transferred to a 96-well plate with 200 µL per well and set in a duplicate hole; then, the plate was measured at OD 562 nm in a preheated microplate reader. A standard curve was defined using Evans blue solution with doubling dilution configurations (100, 50, 25, 12.5, 6.25, 3.12, 1.56, 0.78, and 0 µg/mL), and the content of Evans blue (µg/mL) in the samples was calculated.

### Magnetic resonance imaging (MRI)

MR images were captured using a 9.4-Tesla magnetic resonance imaging scanner. At day 3 after ICH, brain lesion volume and perihematomal edema (PHE) volume were quantified, as previously described 32. To determine the lesion and PHE, scanners used susceptibility-weighted image sequences (repetition time, 30 ms; echo time, 10 ms; field of view, 32 × 32; image matrix, 256 × 256; slice thickness, 0.3 mm) and T2-weighted image sequences (repetition time, 4500 ms; echo time, 65.5 ms; field of view, 28 × 28; image matrix, 256 × 256; slice thickness, 0.5 mm) respectively. Using Medical Image Processing, Analysis, and Visualization software (MIPAV; NIH), the lesion and perihematomal edema volumes were manually marked on each slice, and the areas in all slices were added up and multiplied by section thickness. Two investigators were blinded to the experimental groups when they measured the lesion and PHE.

### Flow cytometry

Flow cytometry was used to measure immune cell subsets and the expression of cytokines and BTK, as previously reported 33. At day 3 following ICH, human peripheral blood samples were collected. Human peripheral blood mononuclear cell suspensions were isolated using lymphoprep (Serumwerk Bernburg, Bernburg, Germany). Cells were centrifuged, then PBS-washed and resuspended in 1% bovine serum albumin (BSA) for antibody staining. At day 3 after ICH, lethal isoflurane anesthesia was used to sacrifice the mice, and the blood, spleen, and brain were subsequently collected for further cell dissociation. Red blood cells in mice blood were destroyed by adding 1 mL of an ammonium-chloride-potassium (ACK) lysis solution to 100 μL of blood and letting it sit at room temperature for 15 minutes. Cells were centrifuged, cleaned in PBS, and then resuspended in 1% BSA to be stained with antibodies. Tissues were ground through 70 μm nylon cell strainers (BD Biosciences, Franklin Lakes, NJ) to separate splenocytes and lysed in ACK lysis buffer. Then, cells were washed in PBS and resuspended in 1% BSA. After pericardial perfusion with cold PBS, mice brain tissues were collected and digested with collagenase IV to form a single cell suspension. The cell pellets were centrifuged at 700 g for 10 min after being resuspended in 5 ml of 30% Percoll (GE Healthcare Bio Science AB, Uppsala, Sweden). On the tube's bottom, cell pellets were collected and suspended in a 1% BSA solution for staining. Cell suspensions were then stained for 30 minutes on ice after the addition of fluorescently-conjugated antibodies. Following surface marker staining, cells were fixed in a fixation buffer for 20 minutes, and following two washes in a permeabilization buffer, intracellular staining was performed. Then, antibodies that target intracellular molecules were introduced and stained overnight at 4 ℃. After being cleaned, cells were suspended in a flow cytometry buffer. Unless otherwise stated, all antibodies were purchased from Biolegend (San Diego, CA, USA). The complete list of antibodies used in flow cytometry was provided in supplementary material (**Table S1**). An LSRFortessa cytometer (BD Biosciences, San Jose, CA) was used to collect the data. Data were analyzed using the FlowJo program (TreeStar).

### Cell culture

BV2 cells were cultured in Dulbecco's Modified Eagle Medium (DMEM, 10566016, Thermo Fisher Scientific) supplemented with 10% fetal bovine serum (FBS, 2053264, Biological Industries) and 1% Penicillin-Streptomycin-Gentamicin Solution (P1410, Solarbio). Cells were seeded on 6-well plates (1×10^7^ cells/ml/well) and maintained in 95% humidified air and 5% CO2 at 37℃ for 2 days. Then, cells were administrated with Vehicle, 40 U/mL thrombin (T8021-1000U, Solarbio), 1uM ibrutinib or 40 U/mL thrombin + 1uM ibrutinib for 24 h 34, 35.

### Serum transaminase measurement and histopathology

An automated biochemical analyzer URIT-8200 (URIT, Guilin, China) was used to evaluate the serum levels of alanine aminotransferase (ALT) and aspartate aminotransferase (AST) after blood samples were collected on days 3, 7, and 14 following ICH induction. The collected liver tissues were treated with 4% paraformaldehyde (PFA), embedded in paraffin blocks and sliced. For histology staining, the liver sections were stained with H&E according to the manufacturer's instructions. Hepatocyte morphology was observed using a light microscope (Olympus, model BX-61).

### Assessments of bleeding time and coagulation time

Following anesthesia, mouse tails were cut off by 2 mm with a scalpel. Then, the truncated tail was immersed in a 15 ml transparent tube containing normal saline at 37°C. The observation period of bleeding time was 20 min. If no bleeding occurred within 30 s, the bleeding was considered stopped. This time was identified as the bleeding time. To assess coagulation time, angular venous blood from anesthetized animals was drawn into 1 mm diameter glass capillaries. The capillary was laid flat on the bench after being completely filled with blood. Then, a small section of the end of the capillary was snapped every 30 s until a fibrin thread appeared. This time was identified as the coagulation time. Both bleeding time and coagulation time were measured at days 1 and 3 after treatment with ibrutinib (10 mg/kg) or vehicle in ICH mice.

### High-performance liquid chromatography (HPLC)

Homogenized brain tissue samples (0.1g) were added into 2 ml 70% methanol, and the mixture was subjected to ultrasonic for 30 min. After the treatment, passing the mixture through a 0.22 μm microporous filter membrane. Measure the filtrate on the liquid Chromatograph (Agilent 1260, USA) with DAD detector, chromatographic column (Agilent C18, 4.6 mm × 250 mm × 5 μm). The mobile phase (acetonitrile: water: 0.1% TFA = 42:31:27) flowed at a rate of 1.0 ml/min. The Column temperature was seated at 36℃ with the spectrum at 258nm. Content is calculated according to the formula: W=(C-C_0_) × V × N / m. In the formula, W represents the content of the Ibrutinib in the sample, with the unit of mg/kg, mg/L; C resembles the concentration of Ibrutinib in sample solution, measured in mg/L; C_0_ means the concentration of Ibrutinib in blank control, using the unit of mg/L; V purports the fixed volume with the unit mL; N denotes the dilution factor; while M indicates the sampling amount of the sample, measured in grams and milliliters.

### Statistical methods

Power analysis and sample size were determined using SAS 9.1 software (SAS Institute Inc.), and based on our experience with the respective tests, variability of the assays and inter-individual differences among experimental groups. The experimental design was based on our prior publications with similar mechanistic studies completed in our laboratory 36-38. Animals were randomly assigned to experimental groups, based on the random number generator function in Microsoft Excel. All experiments presented in this study were repeated at least three times. A two-tailed unpaired Student's t-test was used to compare data from two independent groups. For comparison of two or more variables among multiple groups, two-way ANOVA followed by Tukey post hoc test was used. P < 0.05 was considered to be significant. Data were analyzed with GraphPad prism 8.0. All data were presented as mean ± SD.

## Results

### Upregulation of BTK in immune cells following ICH in humans and mice

**Figure 1:**
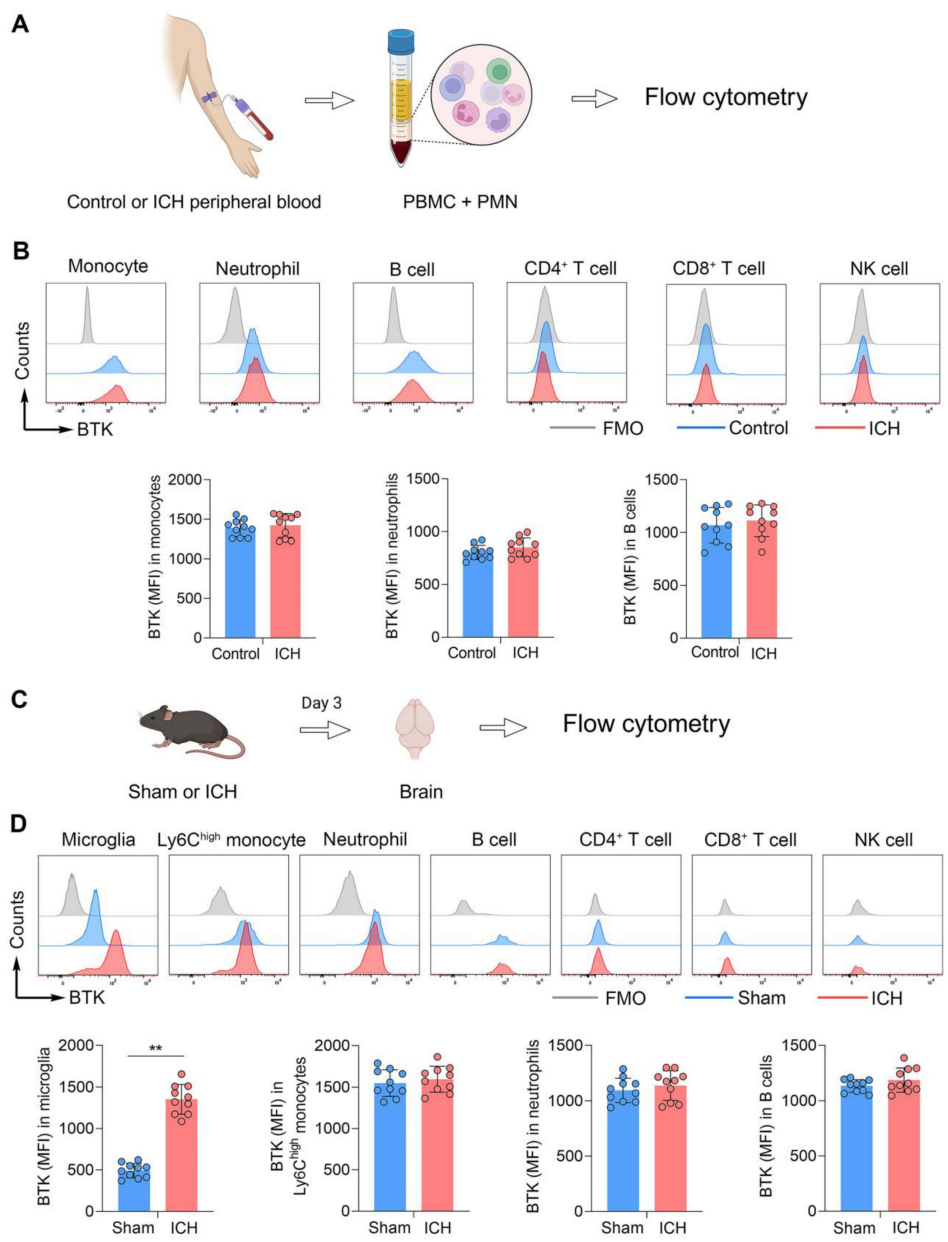
** Upregulation of BTK in humans and mice following ICH. A.** Schematic workflow of the experimental design. **B.** Histograms showing BTK expression across various immune cell subsets (MFI of BTK^+^ monocytes, neutrophils and B cells) in human peripheral blood samples from healthy controls versus patients with ICH. n = 10 per group. ICH was induced in mice by injection of collagenase and mouse brain tissues were collected for flow cytometry analysis at day 3 after surgery.** C.** Schematic workflow of the experimental design. **D.** BTK expression across various immune cell subsets (MFI of BTK^+^ microglia, Ly6C^high^ monocytes, neutrophils and B cells) in sham and ICH mice. n = 10 per group. Data are presented as mean ± SD. **p < 0.01. FMO: fluorescence minus one.

To measure the expression profile of BTK on immune cells, we collected immune cells from the peripheral blood of ICH patients at day 3 after disease onset. Flow cytometry analysis revealed that BTK was mainly expressed in circulating monocytes, neutrophils and B cells (**Figure 1B**). An increase of BTK expression in monocytes, neutrophils and B cells was observed after ICH (**Figure 1B**), although there was no statistical significance. In mice subjected to ICH induced by collagenase injection, we found a dramatic increase of BTK expression in microglia after ICH (**Figure 1D**). Additionally, the expression of BTK was mainly observed in monocytes, neutrophils and B cells (**Figure 1D**). These results demonstrated BTK expression in microglia, monocytes, neutrophils and B cells, together with an increase of BTK in microglia after ICH.

### BTK inhibition reduces neurological deficits and brain edema in two mouse models of ICH

**Figure 2:**
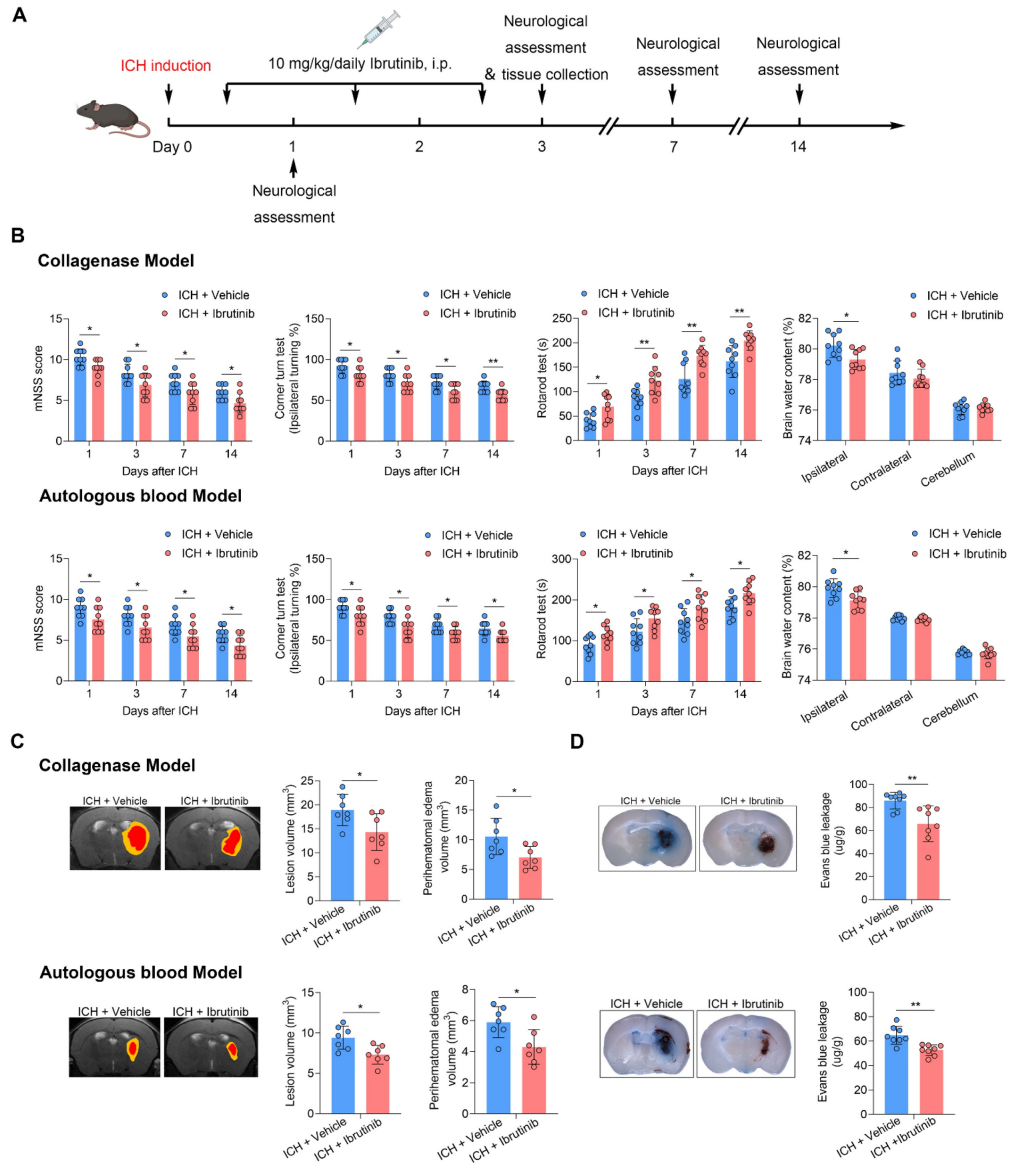
**BTK inhibition attenuates neurological deficits and brain edema. A.** Flow chart of the experimental design. **B.** Neurological tests (mNSS, corner turn test and rotarod test) were performed in mice receiving vehicle or ibrutinib at days 1, 3, 7 and 14 after ICH induced by injection of collagenase or autologous blood. n = 9 per group. Brain water content of ipsilateral, contralateral, and cerebellum was measured in groups of ICH mice receiving vehicle or ibrutinib at day 3 after ICH induced by injection of collagenase or autologous blood. n = 9 per group.** C.** MR images showing lesion volume (in red plus yellow regions) and perihematomal edema volume (in yellow regions) in ICH mice receiving vehicle or ibrutinib (left). Quantification of brain lesion and PHE volume in mice receiving vehicle or ibrutinib at day 3 after ICH induced by injection of collagenase or autologous blood. n = 7 per group. **D.** Histology images and bar graph showing Evans blue leakage in ICH mice receiving vehicle or ibrutinib at day 3 after ICH induced by collagenase injection or autologous blood. n = 8 per group. Data are presented as mean ± SD. *p<0.05, **p<0.01.

Ibrutinib is the first BTK inhibitor approved by FDA for clinical use. To determine the effects of BTK inhibition on ICH injury, we therefore used ibrutinib in two mouse models of ICH induced by injection of autologous blood or collagenase. ICH mice received ibrutinib for 3 consecutive days starting from 12 h after ICH (**Figure 2A**). We found that BTK inhibition reduced neurological deficits and brain water content in in two mouse models of ICH induced by injection of autologous blood or collagenase (**Figure 2B**). The benefits of BTK inhibition against neurological deficits persisted until day 14 after ICH onset (**Figure 2B**). In addition, BTK inhibition also reduced perihematoma edema volume and blood-brain barrier (BBB) disruption in ICH mice (**Figure 2C-D**), which were measured using MRI and Evans blue leakage, respectively.

To determine whether ibrutinib can enter the brain, we measured the concentration of ibrutinib in the brain and plasma using high-performance liquid chromatography (HPLC). We found that ibrutinib can be detected within the brain (**Figure S4C**), which is similar to previous studies 39, 40.

Previous studies have used ibrutinib in mice at doses ranging from 3 to 30 mg/kg 41-45. We also tested the therapeutic effects of ibrutinib in ICH mice using different doses. We found better effects at the dose of 10 mg/kg versus 3 mg/kg. Similar effects were noted at the dose of 10 mg/kg versus 30 mg/kg (**Figure S5B**). In addition, we also examined the effects of a new-generation BTK inhibitor olerabrutinib 46 on ICH injury and found similarly protective effects versus ibrutinib (**Figure S5B**). These results demonstrate that BTK inhibition can reduce neurological deficits and brain edema in ICH.

### BTK inhibition reduces leukocyte infiltration and inflammatory activity of microglia after ICH

**Figure 3:**
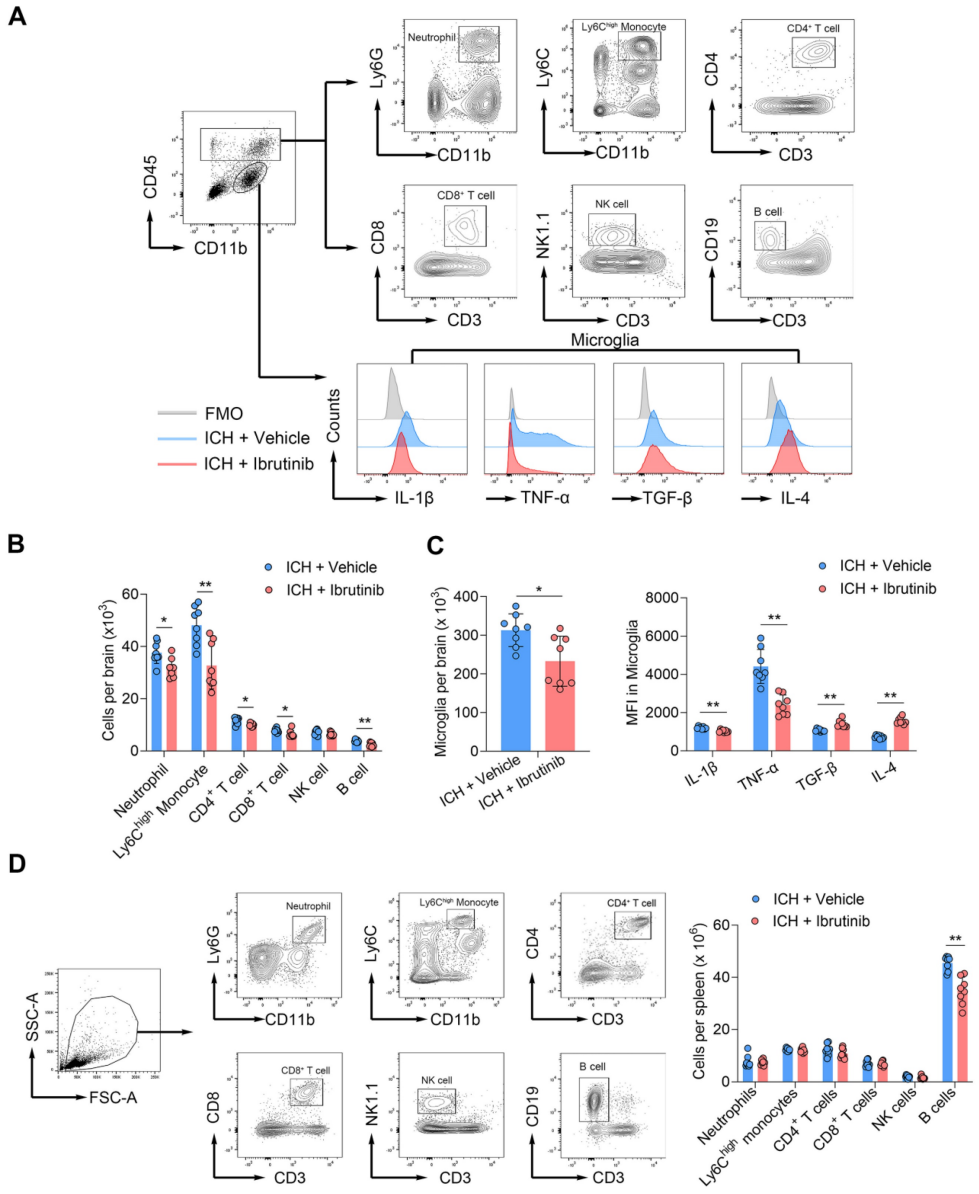
** Effects of ibrutinib on leukocyte infiltration and microglia activity in the brain and leukocytes in the spleen following ICH. A**. Gating strategy of brain-infiltrating neutrophils (CD45^high^CD11b^+^Ly6G^+^), Ly6C^high^ monocytes (CD45^high^CD11b^+^Ly6C^high^), CD4^+^ T cells (CD45^high^CD3^+^CD4^+^), CD8^+^ T cells (CD45^high^CD3^+^CD8^+^), NK cells (CD45^high^CD3^-^NK1.1^+^), B cells (CD45^high^CD3^-^CD19^+^) and microglia (CD11b^+^CD45^int^), as well as their expression of IL-1β, TNF-α, TGF-β and IL-4. **B.** Counts of brain-infiltrating leukocyte subsets in the brains from indicated groups of ICH mice. n = 8 per group. **C.** Counts of microglia and MFI of IL-1β, TNF-α, TGF-β and IL-4 in microglia from indicated groups of ICH mice. n = 8 per group. **D.** Gating strategy of splenic leukocyte subsets, including neutrophils (CD11b^+^Ly6G^+^), Ly6C^high^ monocytes (CD11b^+^Ly6C^high^), CD4^+^ T cells (CD3^+^CD4^+^), CD8^+^ T cells (CD3^+^CD8^+^), NK cells (CD3^-^NK1.1^+^) and B cells (CD3^-^CD19^+^). Counts of leukocyte subsets in the spleens from indicated groups of ICH mice. n = 8 per group. Data are presented as mean ± SD. *p < 0.05, **p < 0.01.

We next sought to test the impact of BTK inhibition on brain inflammation after ICH. Using flow cytometry, we examined leukocyte infiltration and microglia activation in the brains of ICH mice (**Figure 3A**). At day 3 after ICH, we found that ibrutinib treatment reduced the counts of brain-infiltrating neutrophils, Ly6C^high^ monocytes, CD4^+^ T cells, CD8^+^ T cells and B cells (**Figure 3B**), together with a decrease in the counts of microglia and their expression of IL-1β and TNF-α (**Figure 3C**). In contrast, an increase of TGF-β and IL-4 in microglia was observed in ICH mice receiving ibrutinib (**Figure 3C**). Notably, ibrutinib did not significantly alter microglia activity in naive mice (**Figure S6B**). These findings suggest that BTK inhibition can suppress leukocyte infiltration and inflammatory activity of microglia in the ICH brains.

We also measured the counts of splenic immune cell subtypes in ICH mice receiving ibrutinib versus vehicle control. We found that ibrutinib treatment did not significantly alter the counts of splenic neutrophils, Ly6C^high^ monocytes, CD4^+^ T cells and CD8^+^ T cells, although did decrease the counts of splenic B cells (**Figure 3D**). This suggests that inhibition of BTK has minimal effects on the number of immune cell subsets in the periphery other than B cells.

### Benefit of BTK inhibition in ICH involves microglia

**Figure 4:**
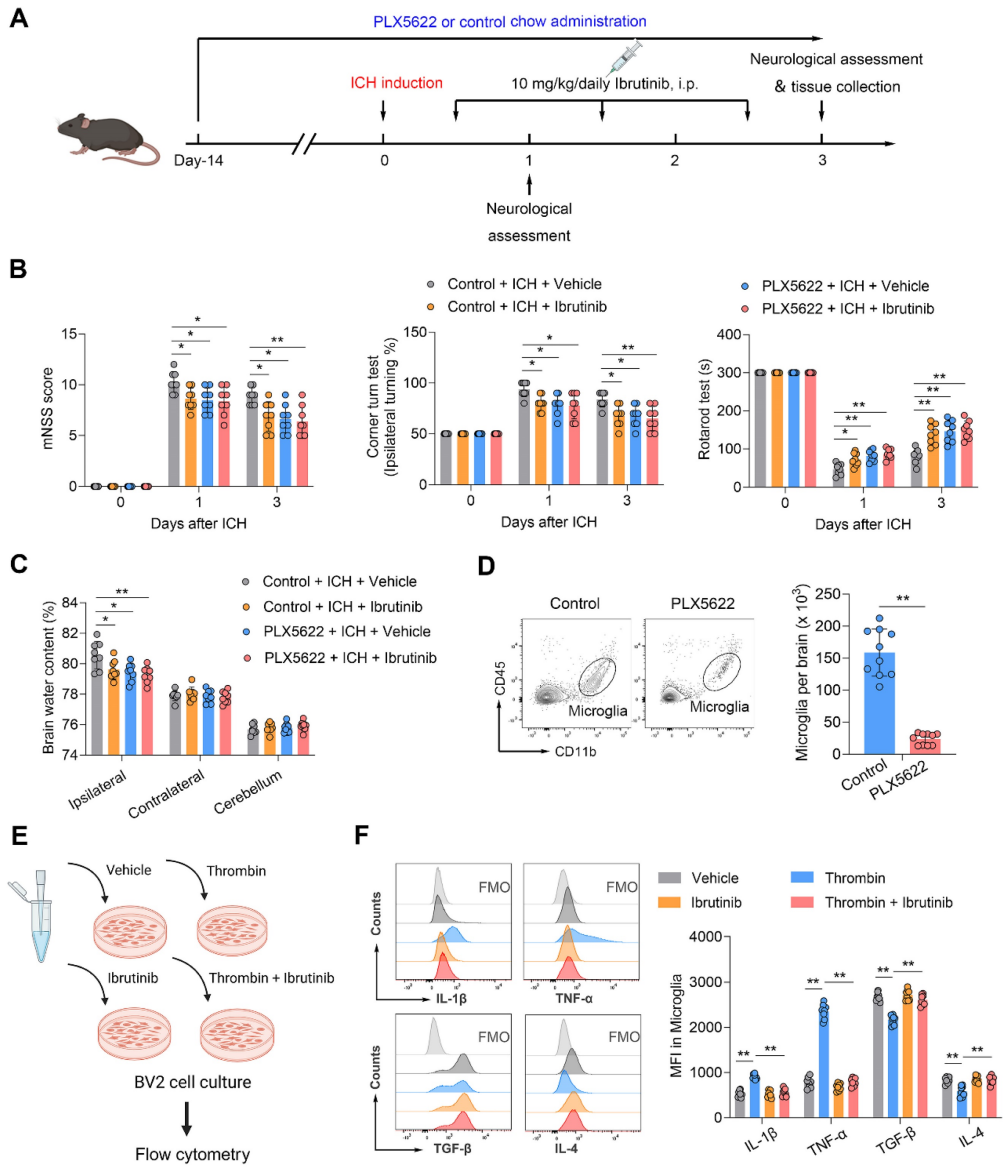
** Microglia contribute to the protective effects of ibrutinib in ICH mice. A.** Flow chart of the experimental design. **B.** Summarized results of mNSS, corner turn test, and rotarod test in mice receiving indicated treatments at day 1 and day 3 after ICH. n = 8 per group. **C.** Brain water content in the ipsilateral, contralateral and cerebellum at day 3 after ICH. n = 8 per group. **D.** Flow cytometry gating strategy and quantification of microglia in the mice receiving a control diet or PLX5622 for 14 days. n = 10 per group. **E.** Schematic workflow of the microglia-like BV2 cell culture experimental design. **F.** Histogram and MFI of IL-1β, TNF-α, TGF-β and IL-4 expression in microglia-like BV2 cells from indicated groups. n = 8 per group. Data are presented as mean ± SD. *P<0.05, **p < 0.01.

As microglia, Gr-1^+^ myeloid cells (neutrophils and monocytes) and B cells express BTK, we sought to understand their contributions to the benefit of BTK inhibition in ICH. Using PLX5622, a colony-stimulating factor 1 receptor (CSF1R) inhibitor, we depleted microglia prior to ICH induction and continued to give ICH mice PLX5622 until the end of experiment (**Figure 4A**). In ICH mice receiving PLX5622, the protective effects of ibrutinib against ICH injury was diminished (**Figure 4B-C**). In addition, PLX5622 administration eliminated > 85% of microglia (**Figure 4D**). These results suggest that microglia contribute to the benefit of BTK inhibition against ICH injury.

### BTK inhibition suppresses thrombin-induced inflammatory activity in microglia-like BV2 cells

To determine whether ibrutinib can modulate microglia activity, we added ibrutinib into the medium of cultured microglia-like BV2 cells that were subjected to thrombin stimulation. Flow cytometry analysis revealed that ibrutinib treatment ablated thrombin-induced production of inflammatory factors such as IL-1β and TNF-α in cultured microglia-like BV2 cells (**Figure 4E-F**). In contrast, the production of immunoregulatory factors such as TGF-β and IL-4 was augmented in cultured microglia-like BV2 cells receiving ibrutinib (**Figure 4F**). Taken together, these results demonstrate that BTK inhibition can suppress the inflammatory activity of microglia in ICH.

### Benefit of BTK inhibition in ICH involves peripheral Gr-1^+^ myeloid cells but not B cells

**Figure 5:**
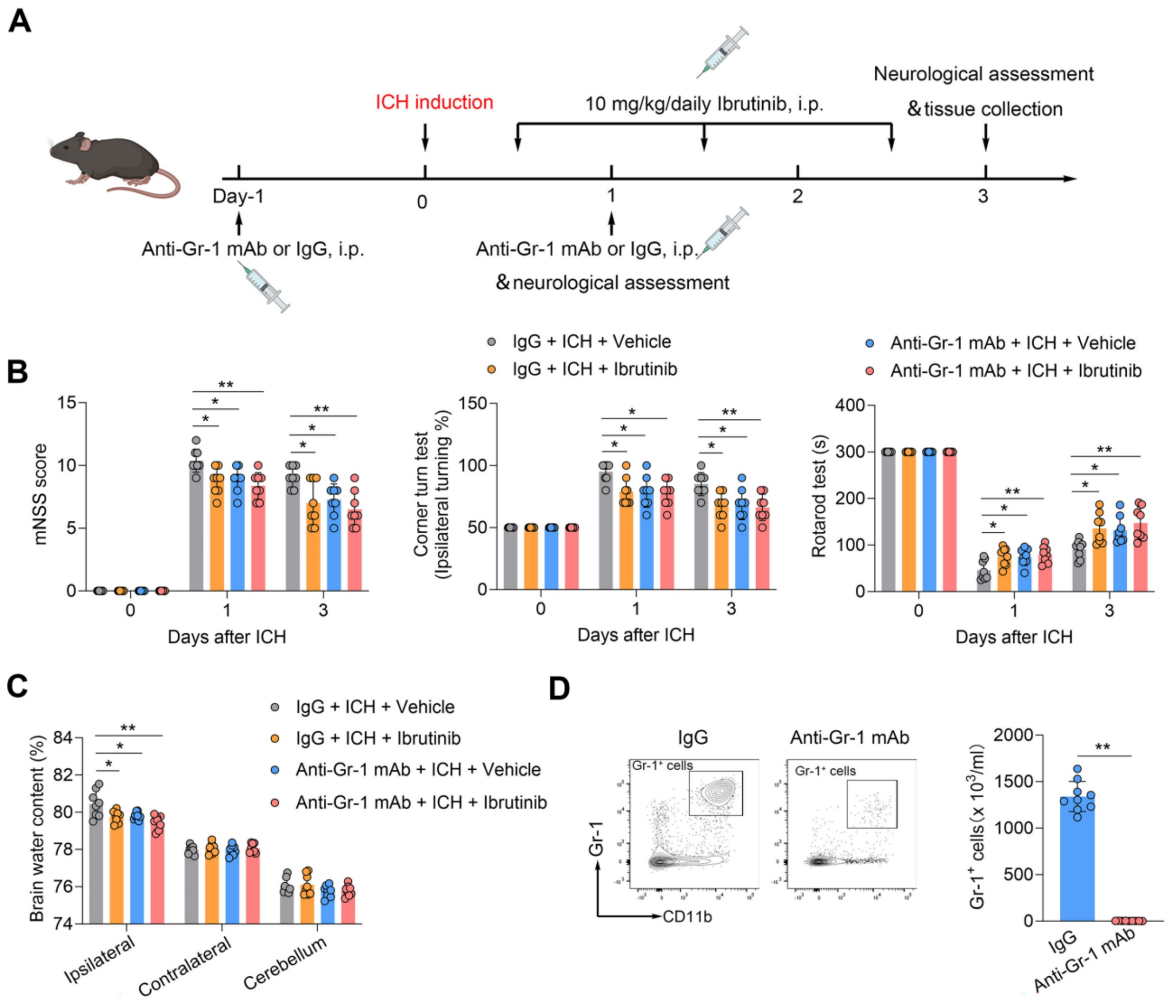
** Gr-1^+^ myeloid cells contribute to the protective effects of ibrutinib in ICH mice. A.** Flow chart of the experimental design.** B.** Summarized results of mNSS, corner turn test, and rotarod test in groups of mice receiving indicated treatments at day 1 and day 3 after ICH. n = 8 per group. **C.** Brain water content in the ipsilateral, contralateral and cerebellum at day 3 after ICH. n = 8 per group. **D.** Flow cytometry gating strategy and quantification of Gr-1^+^ myeloid cells in the peripheral blood in the indicated groups. n = 9 per group. Data are presented as mean ± SD. **p < 0.01.

To test the potential contribution of Gr-1^+^ myeloid cells (monocytes and neutrophils) to the benefit of BTK inhibition against ICH injury, we used anti-Gr-1 mAb (RB6-8C5) to deplete Gr-1^+^ myeloid cells (**Figure 5A**). In ICH mice receiving anti-Gr-1 mAb, we also found diminished benefit of BTK inhibition (**Figure 5B-C**). Anti-Gr-1 mAb eliminated >90% of Gr-1^+^ myeloid cells, which is similar to previous studies 47, 48 (**Figure 5D**). These results suggest that Gr-1^+^ myeloid cells are also involved in the protective effects of ibrutinib against ICH injury.

**Figure 6:**
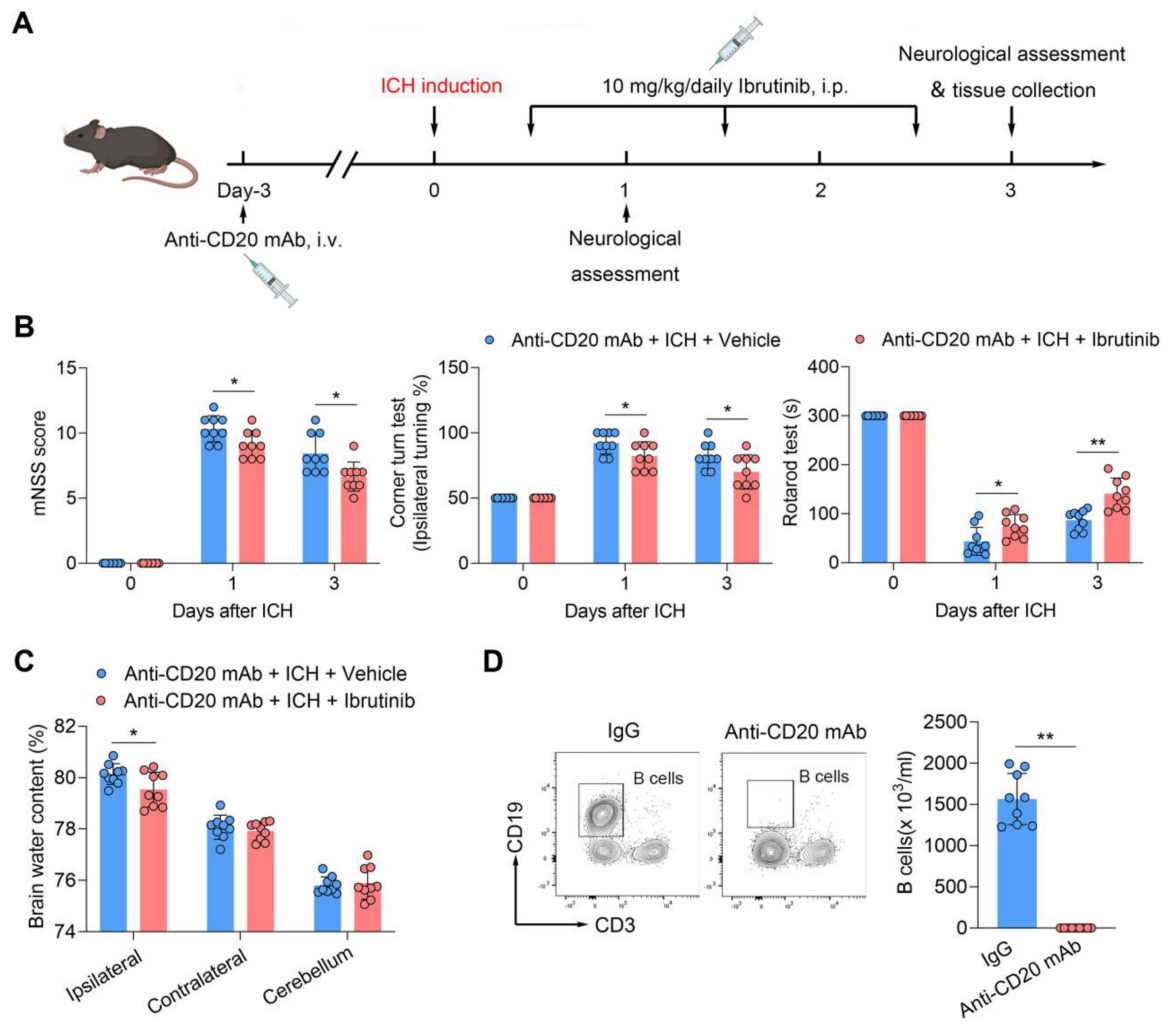
** B cell depletion does not alter the protective effects of ibrutinib in ICH mice. A.** Flow chart of the experimental design.** B.** Summarized results of mNSS, corner turn test, and rotarod test in groups of mice receiving indicated treatments at day 1 and day 3 after ICH. n = 9 per group. **C.** Brain water content in ipsilateral, contralateral, and cerebellum at day 3 after ICH. n = 9 per group. **D.** Flow cytometry gating strategy and quantification of B cells in the peripheral blood in the indicated groups. n = 9 per group. Data are presented as mean ± SD. *p < 0.05, **p < 0.01.

To test whether B cells contribute to the benefit of BTK inhibition in ICH, we depleted B cells using an anti-CD20 mAb (**Figure 6A**). Of note, the benefit of ibrutinib was unaltered in ICH mice receiving anti-CD20 mAb (**Figure 6B-C**), although administration of anti-CD20 mAb eliminated > 90% of B cells (**Figure 6D**), suggesting that B cells are not involved in the protective effects of ibrutinib on brain injury after ICH.

### Short-term exposure to ibrutinib does not cause hepatic injury and bleeding

**Figure 7:**
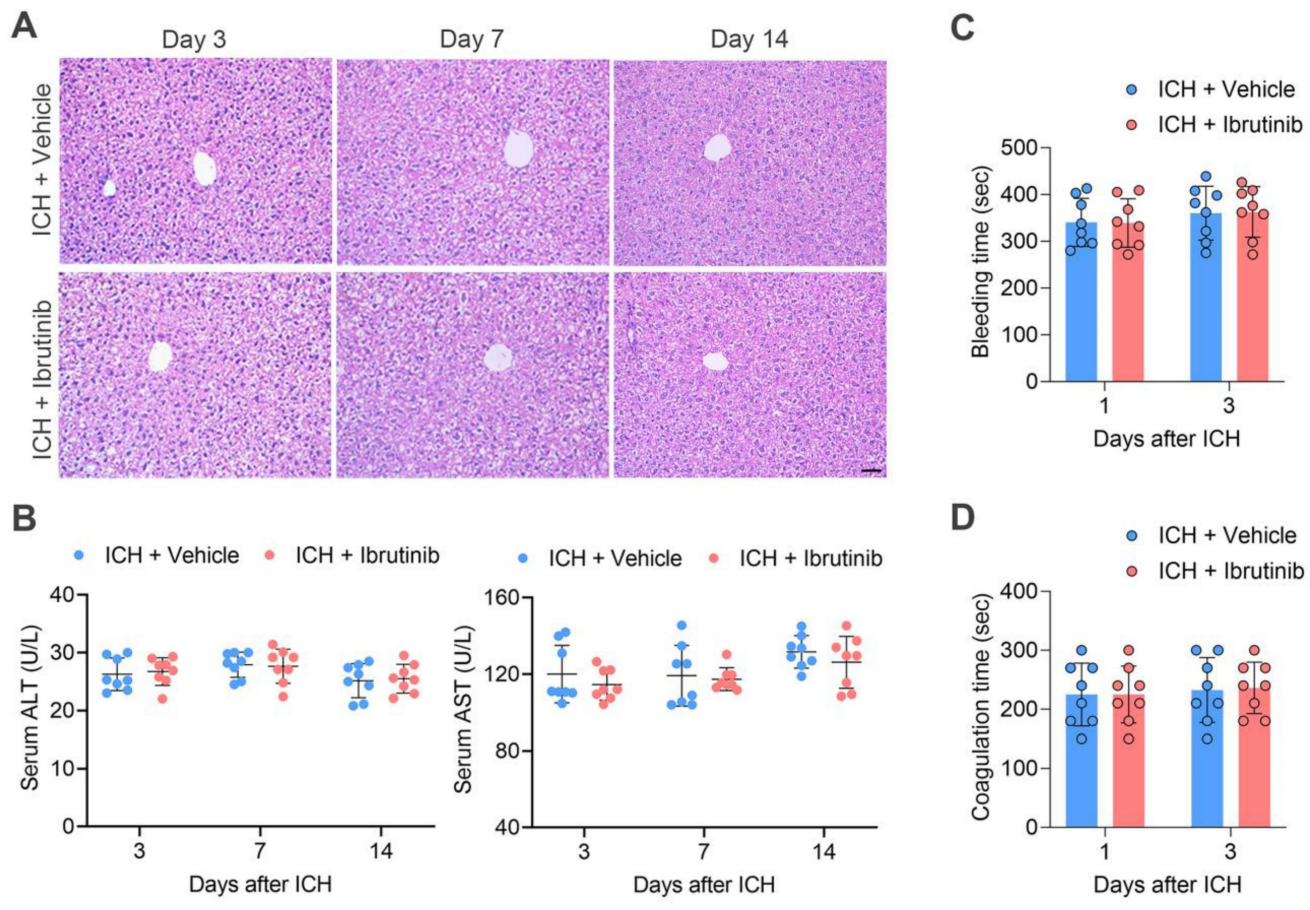
** Effects of ibrutinib treatment on hepatic function and coagulation in ICH mice. A.** H&E staining of liver tissue sections of mice in the indicated groups at days 3, 7 and 14 after ICH. Scare bar: 50 μm. **B.** Serum ALT and AST in the indicated groups of mice at days 3, 7 and 14 after ICH. n = 8 per group. **C.** Bleeding time in the indicated groups of mice at day 1 and day 3 after ICH, n = 8 per group. **D.** Coagulation time in the indicated groups of mice at day 1 and day 3 after ICH, n = 8 per group. Data are presented as mean ± SD.

As long-term use of ibrutinib is known to damage hepatic structure and function, we attempted to test whether short-term use of ibrutinib in this study resulted in hepatic damage, i.e. once daily for 3 consecutive days. For this purpose, we performed H&E staining of liver tissues and found that short-term use of ibrutinib did not alter liver structure (**Figure 7A**). In addition, we measured the circulating alanine transaminase (ALT) and aspartate aminotransferase (AST) in ICH mice receiving ibrutinib. We found that short-term use of ibrutinib did not significantly alter circulating ALT and AST (**Figure 7B**).

We also tested whether short-term use of ibrutinib increases the risk of bleeding, i.e. once daily for 3 consecutive days. Bleeding time and coagulation time were measured respectively. We found that ibrutinib did not significantly alter the bleeding time and coagulation time (**Figure 7C-D**).

## Discussion

Our study demonstrated that the BTK inhibition suppressed neuroinflammation and ICH injury. As documented here, BTK inhibition using ibrutinib significantly attenuated neurological deficits, brain edema, and BBB disruption after ICH in two mouse models of ICH induced by injection of autologous blood or collagenase. In addition, BTK inhibition reduced leukocyte infiltration into the ICH brain and the inflammatory activity of microglia. Notably, the protective effects of BTK inhibition involved microglia and Gr-1^+^ myeloid cells (neutrophils and monocytes) but not B lymphocytes. Together, these results provide novel evidence that BTK inhibition may serve as a viable approach to restrict detrimental neuroinflammation after ICH and improve neurological outcome.

Microglia activation and leukocyte infiltration are known contributors to drive the expansion of PHE after ICH onset 49, 50. As BTK controls the proliferation and effector function of myeloid cells and lymphocytes, it is reasonable to postulate that BTK inhibition can restrict the excessive activation of the inflammatory cascade following ICH. Indeed, we found that BTK inhibition reduced neuroin-flammation and PHE following ICH. Notably, the protective effects of BTK inhibition against ICH injury were diminished after deletion of microglia using a CSF1R inhibitor PLX5622, suggesting that the benefit of BTK inhibition involves its action on microglia. This view is supported by the finding that BTK inhibition effectively suppressed microglial production of inflammatory factors such as IL-1β and TNF-α, and enhanced the immune regulatory activity of microglia as manifested by increased production of IL-4 and TGF-β. In microglia-like BV2 cells 51, we had similar findings. Together with the finding of the upregulation of BTK in microglia after ICH, these results support the notion that modulation of microglia post-ICH contributes to the benefit of BTK inhibition against ICH injury. Other than microglia, PLX5622 also depletes other CSF1R-expressing myeloid cells, including monocyte-derived macrophages 52, 53, perivascular macrophages 54 and meningeal macrophages 55. Future studies are warranted to sort out the specific contributions of microglia to the benefit of BTK inhibition in ICH.

In addition to microglia, we found that Gr-1^+^ myeloid cells (i.e. monocytes and neutrophils) also express BTK. The infiltration of peripheral Gr-1^+^ myeloid cells was restricted following BTK inhibition. Of note, the beneficial effects of ibrutinib in ICH were also diminished by the depletion of Gr-1^+^ myeloid cells using anti-Gr-1 mAb. In line with our findings, previous studies have revealed the detrimental role of monocytes and neutrophils in ICH injury. Together with our findings, these results not only highlight a pivotal role of BTK in control of myeloid cell activation in the setting of ICH, but also demonstrate BTK inhibition as an effective means to restrict harmful neuroinflammation following acute brain injury such as ICH.

BTK governs the maturation and activity of B cells. As BTK is critical for the development and differentiation of B cells, inhibition of BTK can reduce the number activity of B cells in the periphery. In the CNS, BTK is mainly expressed by microglia and BTK inhibition reduce the inflammatory activity of microglia, which is different from the periphery, i.e. BTK inhibition does not significantly alter circulating myeloid cell activity. This discrepancy is likely because microglia are activated within the injured brain following ICH. In contrast, other myeloid cells are not activated. BTK inhibitors may mainly affect activated myeloid cells that depends on the functional status of these cells. Although we found BTK expression in B cells and a decrease of peripheral B cell counts following ICH, the protective effects of BTK inhibition in ICH may not involve B cells. This is supported by the finding that depletion of B cells using anti-CD20 mAb did not alter the protective effects of BTK inhibition against ICH injury. This notion is consistent with previous findings that B cells have no significant contribution to acute ICH injury. Together, these results strengthen the view that microglia and Gr-1^+^ myeloid cells are major contributors to the benefit of ibrutinib against ICH injury.

Recently, BTK inhibitors such as ibrutinib have significant advancement in B-cell malignancies and cancers. The use of BTK inhibitors to treat CNS inflammatory disorders are being actively investigated, including multiple sclerosis (MS) and neuromyelitis optica spectrum disorders (NMOSD). However, a major limiting factor for the clinical use of BTK inhibitors is their adverse effects of liver toxicity after long-term use, i.e. elevations of liver enzymes and lipase 56, 57. In ICH mice, we found that short-term use of ibrutinib did not alter hepatic morphology and function. Previous studies suggest that long-term continuous ibrutinib treatment may result in an increased risk of bleeding such as skin bruises and abrasions, although not severe 58-61. This has been attributed at least partially to the effects of BTK inhibitors on platelet collagen receptor glycoprotein VI and C-type lectin-like receptor 2 62. The underlying mechanisms remain unclear. Although there are no clinical studies were performed using BTK inhibitors such as ibrutinib to treat ICH, BTK inhibitors have been used in several clinical trials to treat autoimmune neurological diseases such as MS (NCT04410978, NCT04410991, NCT04411641) and NMOSD (NCT05356858). These clinical studies have not demonstrated bleeding complications of BTK inhibitors. Notably, BTK inhibitors such as ibrutinib are used in short-term, i.e. days, in the setting of ICH. The short-term use of BTK inhibitors can also reduce its risk of bleeding if any. In this study, we tested the bleeding time and coagulation time in ICH mice receiving ibrutinib. We found that short-term use of ibrutinib did not alter coagulation and bleeding time, as well as hematoma formation, suggesting that short-term use of BTK is safe in the setting of acute ICH. Nevertheless, the clinical use of BTK inhibitors in ICH should be explored with caution and warrant future studies.

In summary, our study demonstrated that inhibition of BTK reduces neuroinflammation and ICH injury. The benefit of BTK inhibition mainly involves its action on microglia and Gr-1^+^ myeloid cells. BTK inhibitors are promising candidates for future design of immune therapies to improve ICH outcome, and await future investigations in clinical trials.

## Supplementary Material

Supplementary figures and table.

## Abbreviations

ICH: intracerebral hemorrhage
CNS: central nervous system
BBB: blood brain barrier
BTK: bruton's tyrosine kinase
FMO: fluorescence minus one
IL-1β: interleukin-1β
TNF-α: tumor necrosis factor-α
TGF-β: transforming growth factor-β
IL-4: interleukin-4
PBMC: peripheral blood mononuclear cell
PMN: polymorphonuclear
ACK: ammonium-chloride-potassium
DMSO: dimethyl sulfoxide
PBS: phosphate-buffered saline
BSA: bovine serum albumin
PFA: paraformaldehyde
H&E: hematoxylin-eosin staining
ALT: alanine transaminase
AST: aspartate aminotransferase
mNSS: modified neurological severity score
