# Supplementary material for: Inhibition of Bruton's tyrosine kinase restricts neuroinflammation following intracerebral hemorrhage
Source: Theranostics. 2025 Jan 1;15(2):494–508. doi: 10.7150/thno.101024 (PMC11671375; doi:10.7150/thno.101024)
Supplement: Supplementary file 1 — Supplementary figures and table. [file thnov15p0494s1.pdf]

Supplementary material

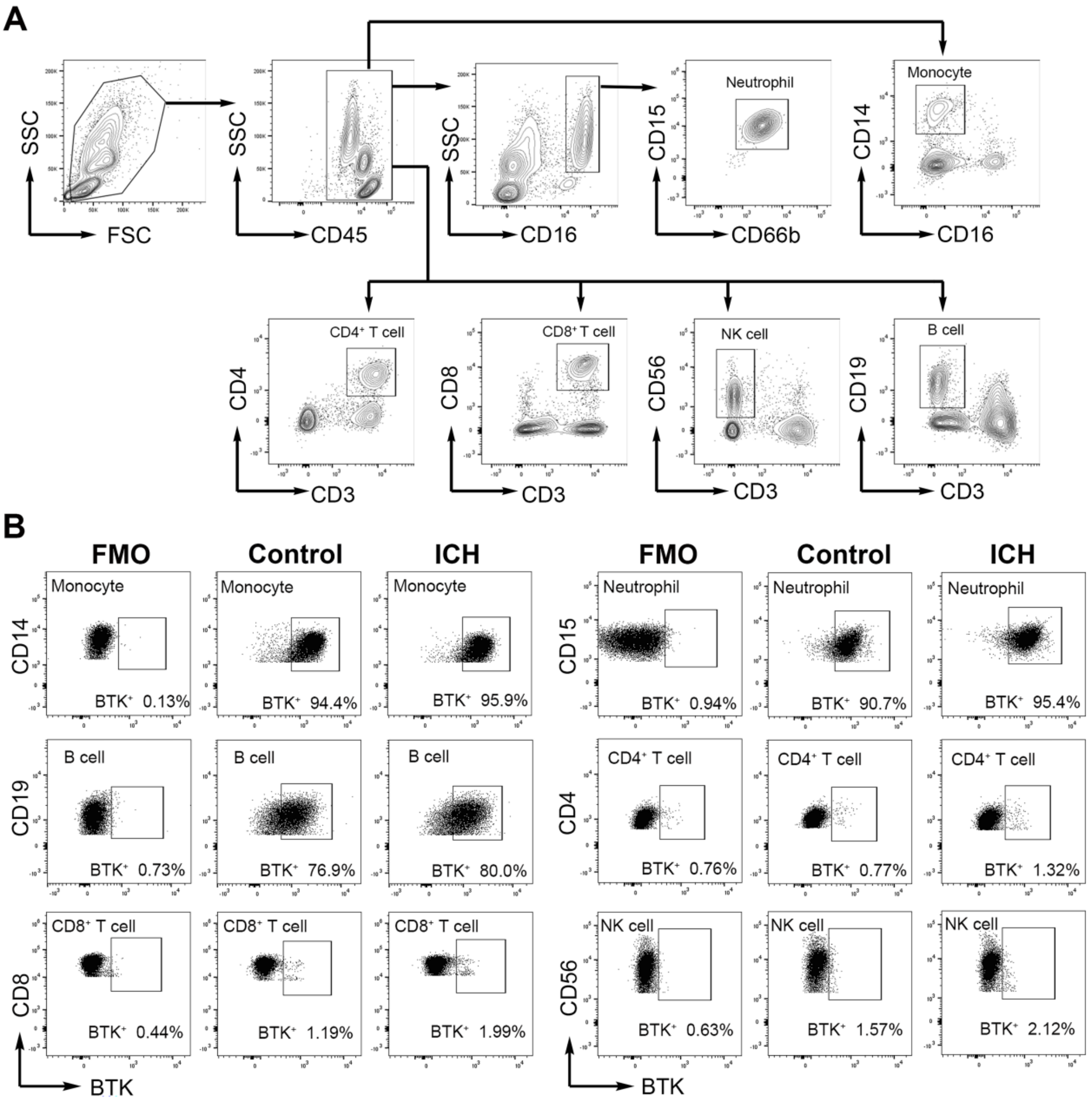

Figure S1. Flow cytometry gating strategy of BTK in Human peripheral blood immune cells.

A. Flow cytometry gating strategy of human circulating neutrophils ($CD45^+CD16^+CD66b^+CD15^+$), monocytes ($CD45^+CD16^+CD14^+$), $CD4^+$ T cells ($CD45^+CD3^+CD4^+$), $CD8^+$ T cells ($CD45^+CD3^+CD8^+$), NK cells ($CD45^+CD3^-CD56^+$) and B cells ($CD45^+CD3^-CD19^+$). **B.** Dot plots showing the expression of BTK in peripheral blood immune cells from patients with ICH or controls.

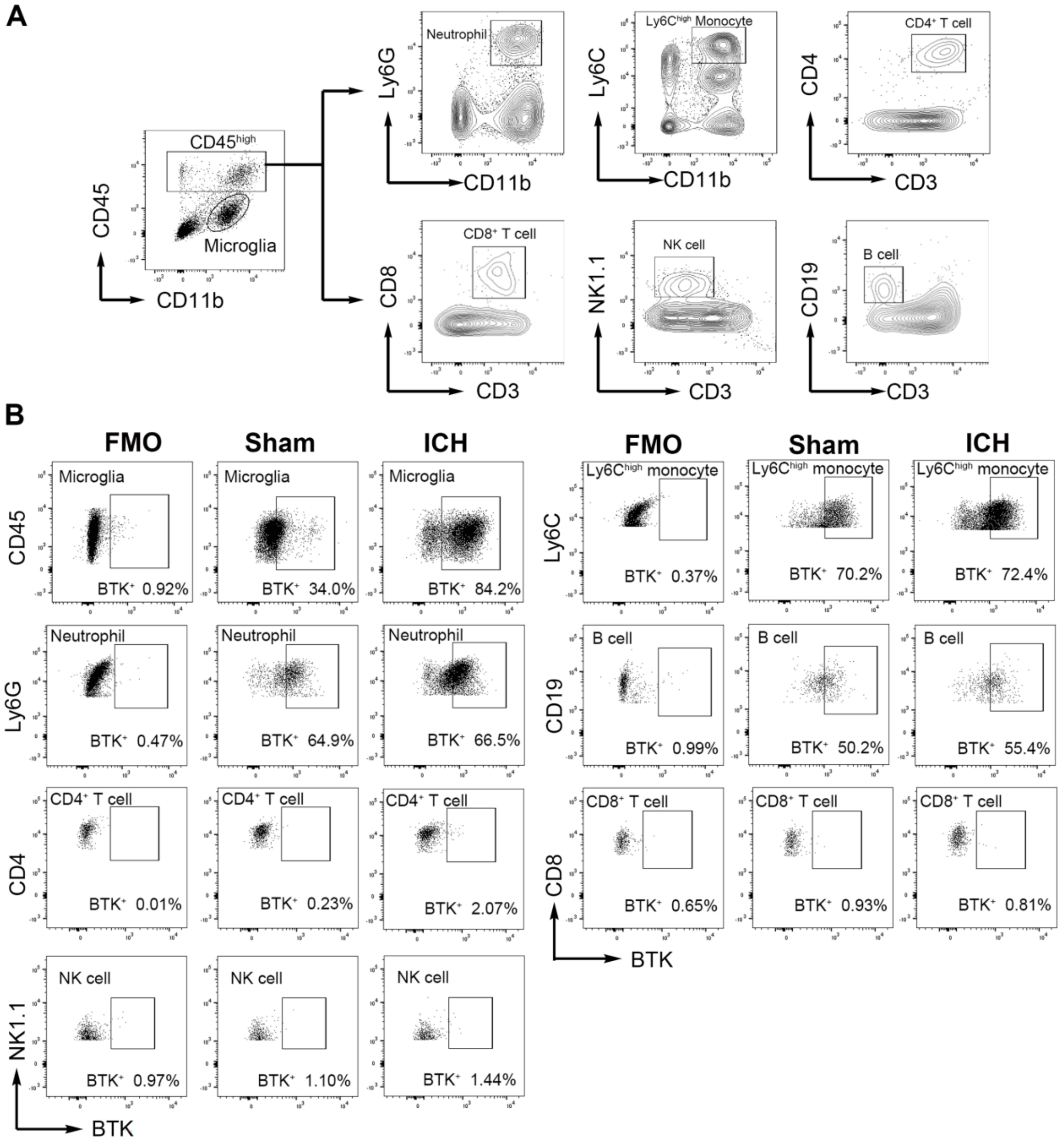

Figure S2. Flow cytometry gating strategy of BTK in mice brain immune cells.

A. Flow cytometry gating strategy of mouse microglia ($CD11b^{+}CD45^{int}$), neutrophils ($CD45^{high}CD11b^{+}Ly6G^{+}$), $Ly6C^{high}$ monocytes ($CD45^{high}CD11b^{+}Ly6C^{high}$), $CD4^{+}$ T cells ($CD45^{high}CD3^{+}CD4^{+}$), $CD8^{+}$ T cells ($CD45^{high}CD3^{+}CD8^{+}$), NK cells ($CD45^{high}CD3^{-}NK1.1^{+}$) and B cells ($CD45^{high}CD3^{-}CD19^{+}$). **B.** Dot plots of the expression of BTK in brain immune cells of sham or ICH mice.

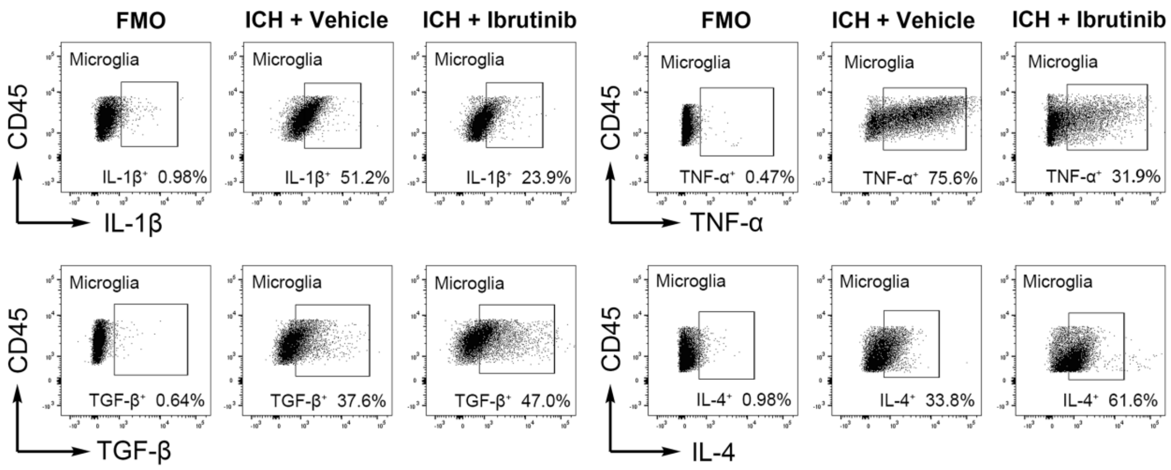

Figure S3. Flow cytometry dot plots showing cytokine expression profile in microglia from indicated groups of mice.

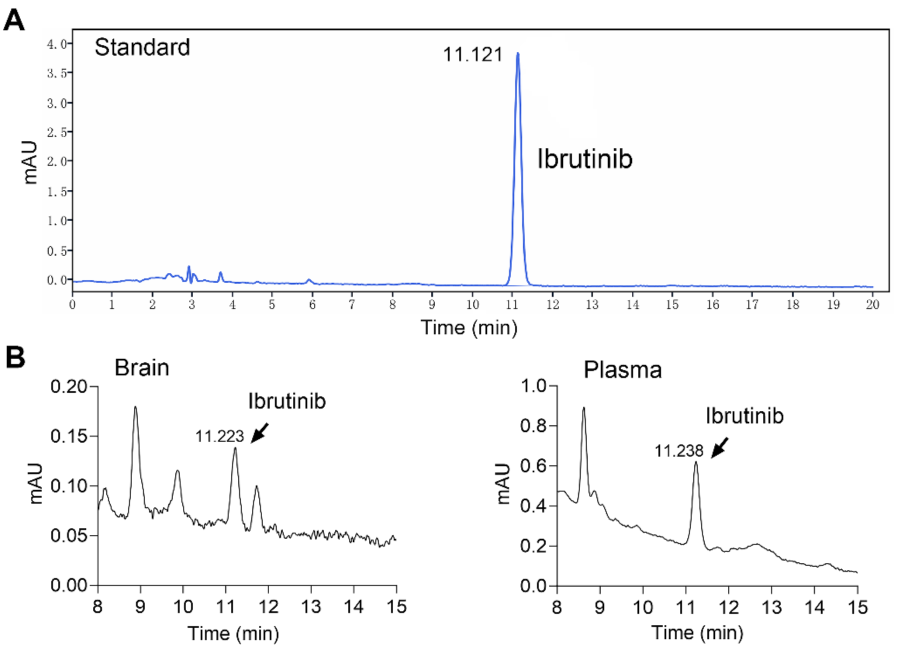

C

Concentrations of ibrutinib in mice brain and plasma samples

	A	B	C	D	E	F	G
Brain samples (ug/mg)	0.27	0.17	0.26	0.23	0.26	0.22	0.45
Plasma samples (ug/ml)	0.98	0.69	1.10	0.78	0.46	0.36	0.33

Figure S4. Ibrutinib distribution in the brain and periphery in ICH mice.

ICH was induced in mice by injection of collagenase. Mice received daily intraperitoneal (i.p.) injections of ibrutinib (10 mg/kg) for 3 consecutive days starting from 12 h after ICH induction. At 1 h after ibrutinib administration on day 3, brain and plasma samples were collected to measure ibrutinib concentrations by high-performance liquid chromatography (HPLC). **A.** Chromatogram of standard ibrutinib. **B.** Chromatograms of brain and plasma samples. **C.** Concentrations of ibrutinib in mice brain and plasma samples.

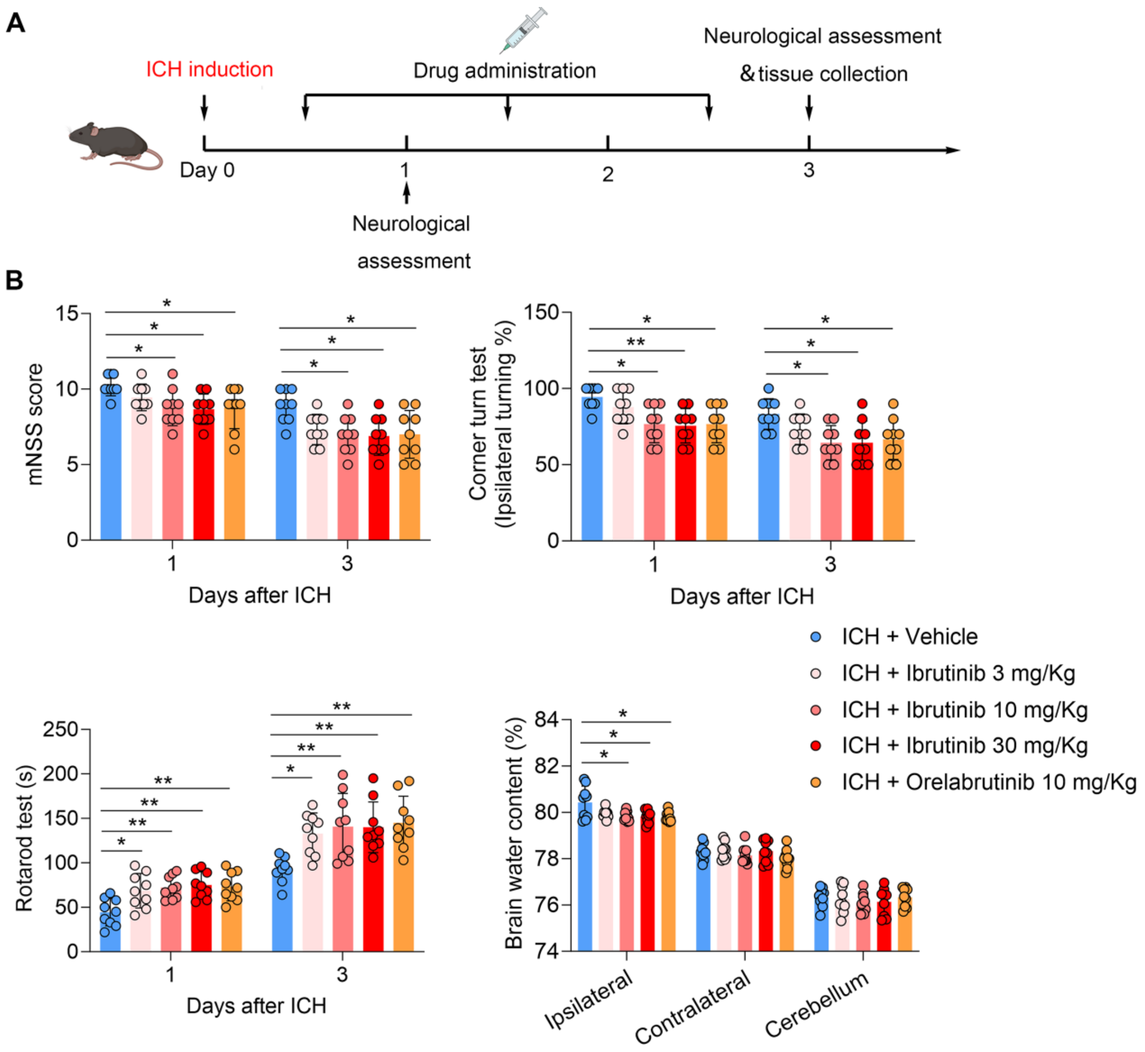

Figure S5. Effects of different doses of ibrutinib and orelabrutinib on ICH injury in mice.

ICH was induced in mice by injection of collagenase. **A.** Flow chart illustrates drug administration and experimental design. Mice received daily i.p. injections of ibrutinib (3, 10, 30 mg/kg), orelabrutinib (10 mg/kg) or an equal volume of vehicle for 3 consecutive days starting from 12 h after ICH induction. **B.** Neurological tests (mNSS, corner test and rotarod test) were performed in indicated groups of mice at day 1 and day 3 after ICH induction. n = 9 per group. Brain water content was measured in indicated groups of ICH mice at day 3 after ICH. n = 9 per group. Data are presented as mean ± SD. *p<0.05, **p<0.01.

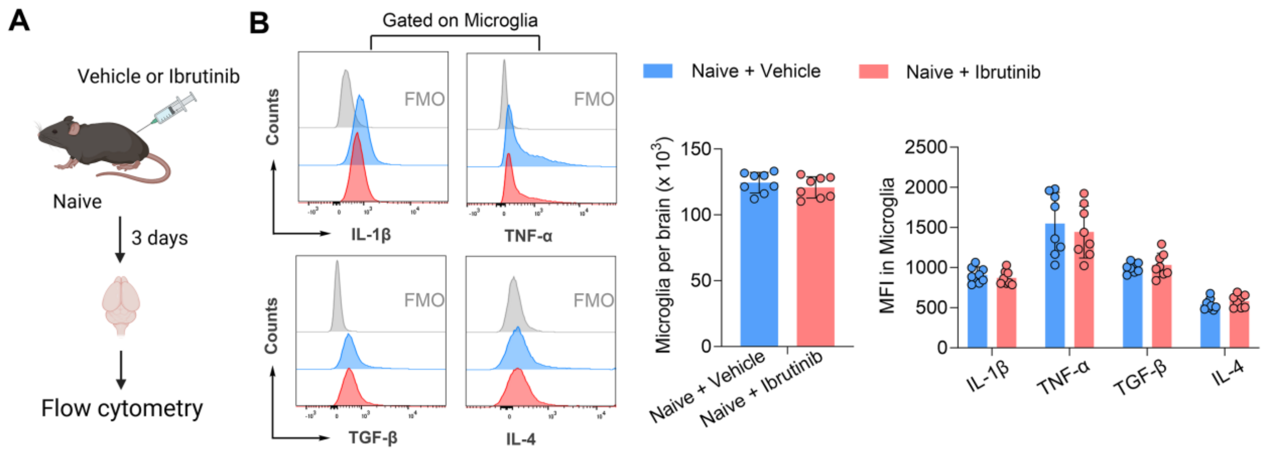

Figure S6. Effects of ibrutinib on microglia in naïve mice.

A. Schematic workflow of experimental design. Naïve mice received daily intraperitoneal (i.p.) injections of ibrutinib (10 mg/kg) or an equal volume of vehicle for 3 consecutive days. Brain tissues were collected on day 3 after ICH for flow cytometry analysis. **B.** Histograms and bar graphs showing the counts of microglia and their expression of indicated cytokines (IL-1β, TNF-α, TGF-β and IL-4). n = 8 per group. Data are presented as mean ± SD.

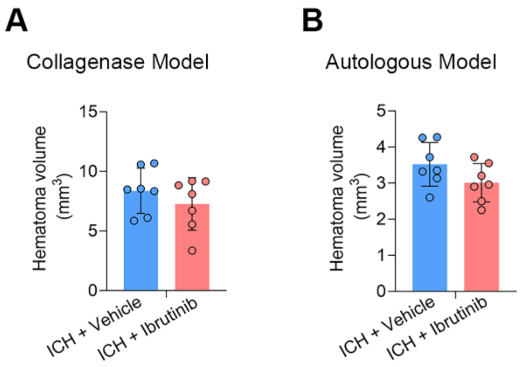

Figure S7. Hematoma volume in ICH mice receiving ibrutinib.

ICH was induced in mice by injection of collagenase or autologous blood. Mice received daily intraperitoneal (i.p.) injections of ibrutinib (10 mg/kg) or an equal volume of vehicle for 3 consecutive days starting from 12 h after ICH induction. MR images were used to measure hematoma volume (in red regions) in ICH mice receiving vehicle or ibrutinib. **A.** Quantification of brain hematoma volume in mice receiving vehicle or ibrutinib at day 3 after ICH induced by injection of collagenase. n = 7 per group. **B.** Quantification of brain hematoma volume in mice receiving vehicle or ibrutinib at day 3 after ICH induced by injection of autologous blood. n = 7 per group. Data are presented as mean ± SD.

Table S1. List of antibodies used in this study.

Antibodies used in flow cytometry	Source	Identifier
PE/Cyanine7 anti-mouse/human CD11b (Clone M1/70)	BioLegend	Cat# 101216; RRID: AB_312799
FITC anti-mouse CD45 (Clone S18009F)	BioLegend	Cat# 157214; RRID: AB_2894427
APC/Cyanine7 anti-mouse Ly-6G (Clone 1A8)	BioLegend	Cat# 127624; RRID: AB_10640819
PE anti-mouse Ly-6C (Clone HK1.4)	BioLegend	Cat# 128008; RRID: AB_1186132
PerCP/Cyanine5.5 anti-mouse CD3 (Clone 17A2)	BioLegend	Cat# 100218; RRID: AB_1595492
PE anti-mouse CD4 (Clone GK1.5)	BioLegend	Cat# 100408; RRID: AB_312693
Brilliant Violet 421™ anti-mouse CD8a (Clone 53-6.7)	BioLegend	Cat# 100738; RRID: AB_11204079
APC anti-mouse CD19 (Clone 1D3/CD19)	BioLegend	Cat# 152410; RRID: AB_2629839
PE/Cyanine7 anti-mouse CD19 (Clone 1D3/CD19)	BioLegend	Cat# 152418; RRID: AB_2927870
APC anti-mouse NK-1.1 (Clone S17016D)	BioLegend	Cat# 156506; RRID: AB_2876525
PE anti-mouse LAP (TGF-β1) (Clone TW7-16B4)	BioLegend	Cat# 141404; RRID: AB_10720867
Alexa Fluor® 647 anti-mouse TNF-α (Clone MP6-XT22)	BioLegend	Cat# 506314; RRID: AB_493330
PerCP/Cyanine5.5 anti-mouse IL-4 (Clone 11B11)	BioLegend	Cat# 504124; RRID: AB_2561565

PE anti-mouse Ly-6G/Ly-6C (Gr-1) (Clone RB6-8C5)	BioLegend	Cat# 108408; RRID: AB_313373
FITC anti-human CD45 (Clone HI30)	BioLegend	Cat# 304038; RRID: AB_2562050
APC/Cyanine7 anti-human CD45 (Clone HI30)	BioLegend	Cat# 304014; RRID: AB_314402
PerCP anti-human CD16 (Clone 3G8)	BioLegend	Cat# 302030; RRID: AB_940380
APC anti-human CD15 (SSEA-1) (Clone HI98)	BioLegend	Cat# 301908; RRID: AB_314200
PE/Cyanine7 anti-human CD66b (Clone G10F5)	BioLegend	Cat# 305116; RRID: AB_2566605
Brilliant Violet 421™ anti-human CD14 (Clone 63D3)	BioLegend	Cat# 367144; RRID: AB_2810580
APC anti-human CD3 (Clone OKT3)	BioLegend	Cat# 367144; RRID: AB_2810580
APC/Cyanine7 anti-human CD19 (Clone HIB19)	BioLegend	Cat# 302218; RRID: AB_314248
PerCP anti-human CD4 (Clone RPA-T4)	BioLegend	Cat# 300528; RRID: AB_893321
PE/Cyanine7 anti-human CD8 (Clone SK1)	BioLegend	Cat# 344712; RRID: AB_2044008
PE anti-human CD56 (NCAM) (Clone 5.1H11)	BioLegend	Cat# 362508; RRID: AB_2563925
Ultra-LEAF™ Purified anti-mouse CD20 (Clone SA271G2)	BioLegend	Cat# 152116; RRID: AB_2629619
Ultra-LEAF™ Purified anti-mouse Ly-6G/Ly-6C (Gr-1) (Clone RB6-8C5)	BioLegend	Cat# 108453; RRID: AB_2616681

APC anti-mouse IL-1β (Pro-form) (Clone NJTEN3)	Thermo Fisher Scientific	Cat# 17-7114-80; RRID: AB_10670739
Anti-mouse BTK (Clone EPR20445)	abcam	Cat# ab208937
Alexa Flour™ 405 Goat anti-Rabbit IgG(H+L) cross-adsorbed secondary antibody	Thermo Fisher Scientific	Cat# A-31556; RRID: AB_221605
Phosflow™ Alexa Flour® 647 Mouse anti-human BTK	BD Biosciences	Cat# 558528; RRID: AB_647112
